# Supplementary material for: Prompt-guided and multimodal landscape scenicness assessments with vision-language models
Source: PLoS One. 2024 Sep 30;19(9):e0307083. doi: 10.1371/journal.pone.0307083 (PMC11441650; doi:10.1371/journal.pone.0307083)
Supplement: S1 Appendix — (PDF) [file pone.0307083.s001.pdf]

Appendix S1. SigLIP results

This section shows the results for experiments performed using SigLIP embeddings. Performance across all experiments is similar to CLIP embeddings, with the only notable difference being the best prompt contexts emerging from contrastive prompts.

Table S1. Late ensembling results with SigLIP embeddings. Better metric performances are shown in bold.

Method	voters	Total Prompts	RMSE	R^2	τ
**Early**	27	137	**2.751**	0.515	0.343
**Late**					
>= 2 prompts	18	129	2.73	**0.702**	**0.501**
>= 5 prompts	10	105	2.40	0.653	**0.46**
>= 8 prompts	5	74	2.72	0.66	0.472
>= 10 prompts	3	49	**3.03**	**0.628**	0.442

Fig S1. SigLIP metric comparisons

Fig S2. SigLIP zero-shot contrastive prompting performance
