# Supplementary material for: Prompt-guided and multimodal landscape scenicness assessments with vision-language models
Source: PLoS One. 2024 Sep 30;19(9):e0307083. doi: 10.1371/journal.pone.0307083 (PMC11441650; doi:10.1371/journal.pone.0307083)
Supplement: S2 Appendix — (PDF) [file pone.0307083.s002.pdf]

Appendix S2. Most-activated prompts

This section discusses the most commonly activated prompts for each Level-2 CORINE land cover class. Table S1 displays the land cover class names, while Table S2 shows the activations per class. The model generally activates relevant prompts for each land cover class, though on some fine-grained classes, such as inland and marine water, it does not activate the most relevant ones.

Table S1. Land cover classes considered in this paper.

Category	ID	Class Name
**Artificial surfaces**		
	1.1	Urban fabric
	1.2	Industrial, commercial, and transport units
	1.3	Mine, dump and construction sites
	1.4	Artificial, non-agricultural vegetated areas
**Agricultural areas**		
	2.1	Arable land
	2.2	Permanent crops
	2.4	Heterogeneous agricultural areas
**Forests and semi-natural areas**		
	3.1	Forest
	3.2	Shrub and/or herbaceous vegetation associations
	3.3	Open spaces with little or no vegetation
**Wetlands**		
	4.1	Inland wetlands
	4.2	Coastal wetlands
**Water bodies**		
	5.1	Inland waters
	5.2	Marine waters

Table S2. Most-activated prompts for each level-2 CORINE land cover class.

LC Class	Most Activated Prompts
**1.1**	(0.146) A Victorian building along an asphalt road<br>(0.104) An urban Victorian building<br>(0.067) Suburban area with regular rows of terraced houses
**1.2**	(0.129) A Victorian building along an asphalt road<br>(0.064) Suburban area with regular rows of terraced houses<br>(0.05) road with hedges alongside
**1.3**	(0.146) Areas of grassland and agricultural fields (farmland), bordered or separated by low stone walls<br>(0.119) A huge plain area with fields and open sky<br>(0.042) Hilly outlooks over irregular square patches of agricultural land
**1.4**	(0.064) Suburbs of London with big villas and wild gardens with beautiful trees<br>(0.061) Areas of grassland and agricultural fields (farmland), bordered or separated by low stone walls<br>(0.057) A Victorian building along an asphalt road
**2.1**	(0.191) Areas of grassland and agricultural fields (farmland), bordered or separated by low stone walls<br>(0.099) Hilly outlooks over irregular square patches of agricultural land<br>(0.093) road with hedges alongside
**2.2**	(0.115) Areas of grassland and agricultural fields (farmland), bordered or separated by low stone walls<br>(0.111) road with hedges alongside<br>(0.089) Rural village with typical red brick houses
**2.4**	(0.252) Areas of grassland and agricultural fields (farmland), bordered or separated by low stone walls<br>(0.074) road with hedges alongside<br>(0.051) Hilly outlooks over irregular square patches of agricultural land
**3.1**	(0.118) Areas of grassland and agricultural fields (farmland), bordered or separated by low stone walls<br>(0.108) A moor with low vegetation in the mountains<br>(0.062) A narrow road inside a forest
**3.2**	(0.224) A moor with low vegetation in the mountains<br>(0.202) Areas of grassland and agricultural fields (farmland), bordered or separated by low stone walls<br>(0.099) A bog with grass surrounded by a mountain
**3.3**	(0.23) A moor with low vegetation in the mountains<br>(0.13) A bog with grass surrounded by a mountain<br>(0.093) Areas of grassland and agricultural fields (farmland), bordered or separated by low stone walls
**4.1**	(0.144) Areas of grassland and agricultural fields (farmland), bordered or separated by low stone walls<br>(0.13) Hilly outlooks over irregular square patches of agricultural land<br>(0.071) A huge plain area with fields and open sky
**4.2**	(0.235) A moor with low vegetation in the mountains<br>(0.232) A bog with grass surrounded by a mountain<br>(0.123) Areas of grassland and agricultural fields (farmland), bordered or separated by low stone walls
**5.1**	(0.076) Sandy beaches<br>(0.056) sea shore<br>(0.056) Areas of grassland and agricultural fields (farmland), bordered or separated by low stone walls
**5.2**	(0.15) A loch with mountains in a forest<br>(0.123) A lake in the highlands surrounded by hills<br>(0.087) A lake in the highlands surrounded by mostly green mountains
