# Supplementary material for: Prompt-guided and multimodal landscape scenicness assessments with vision-language models
Source: PLoS One. 2024 Sep 30;19(9):e0307083. doi: 10.1371/journal.pone.0307083 (PMC11441650; doi:10.1371/journal.pone.0307083)
Supplement: Attachment — Submitted filename: response_letter.pdf [file pone.0307083.s003.pdf]

Prompt-guided and multimodal landscape scenicness assessments with vision-language models

Plos One: Review response letter
by A. Levering and co-authors

May 21, 2024

We would like to thank the editor and the reviewers for their insightful comments. We have responded to all comments one by one below, and modified the manuscript accordingly. Changes to the manuscript include comparisons to SigLIP embeddings in Appendix 1, an extra experiment to analyze the relevancy of activated prompts, and a reduced emphasis on CLIP. Changes in the manuscript are reported in blue.

Reviewer 1

1. **Statistical analysis for Figure 7, Table 2, and Figure 8 is needed for a comprehensive understanding of the performance in different settings. This is crucial to support the conclusions, especially since the size of the testing samples is small.**

   Thank you for the suggestion, we agree that it is necessary to evaluate the effects of the sample sizes on our conclusions. We ran Welch's t-tests for the 25-sample metric comparisons of Figure 7, and the ensembling results of Figure 8, as they are calculated over small sample sizes. Out of these, the only ranking differences that were found to be statistically insignificant ($p > 0.05$) were between land cover classes 1.2 and 1.3, and 2.1 and 2.2 of Figure 9. As the metrics of Table 2 are calculated by

prediction value comparisons with the entire SON dataset with over 210′000 samples, we did not run a t-test for this table.

2. **The manuscript seems to imply that late ensembling involves multiple score fusions, including intra-rater scoring with multiple prompts and inter-rater scoring. Clarification on why these differences matter, their computational complexities, and the benefits of the preferred method would be helpful.**

   We have added additional clarifications in the text to clarify the rationale behind the ensembling methods. To further expand on the implications of early versus late ensembling, we have added an additional experiment, which tests if prompts activate on expected samples, in order to study why early ensembling underperforms compared to late ensembling, and to justify the choice for each method. Regarding the computational complexity, it is fortunately very light as it only involves a few matrix calculations, and both methods run in seconds on pre-calculated embeddings, even on larger datasets. We have added some details about the complexity in the discussion section.

3. **Comparing different backbones in Figure 6 may not be fair for assessing performance capability in few-shot or zero-shot learning. The model capabilities could have performance gaps due to varying backbones and pretrained weights.**

   We agree that the comparison to the baseline was not representative, and as such we have fine-tuned a pre-trained ViT-14/L model with CLIP pre-trained weights. It achieved slightly better metrics on the test set, and as such we have replaced the ConvNeXt baseline in Figure 6. Thank you for the suggestion.

Reviewer 2

1. **The main issue of this manuscript is the novelty. This kind of few-shot setting on CLIP has been widely studied in previous works [13, 28]. Apart from CLIP, recent multimodal large language models (MLLMs), such as GPT-4V [i] and LLaVA [ii], have shown stronger performance. It will be better to add experiments based on them to make this paper more comprehensive.**

We thank the reviewer for their suggestion to consider the use of MLLM models. Implementations such as LLaVa indeed showcase promising capabilities of downstream tasks. However, as it is not straightforward to compare separately acquired vision and text embeddings from such models, it would become computationally expensive to move towards extracting joint embeddings for every image and prompt combination. The use of VLMs rather than MLLMs make the analyses fast and computationally light, which is better aligned with the paper's message of accessible dataset creation and processing for landscape aesthetic analyses. To address the request for comparisons with more recent methods, we have included experiments using SigLIP embeddings [1], a more recent method for pre-training VLMs. However, we did not observe a consistent improvement in performance when using these embeddings. Nevertheless, we report the results in Appendix 1 of the paper. As can be seen, the model performs better on some tasks and in some settings (in bold, when the result improves over the CLIP baseline), but are worse in others (not in bold).

2. **In L216, it mentions that the CLIP encoder is frozen even for few-shot experiments. I am wondering why it should not be updated. From my understanding, landscape images are mostly similar and require more detailed visual features to extract useful patterns for scenicness prediction. However, CLIP is not pre-trained for this fine-grained scenario, where a fixed CLIP encoder may not extract sufficient features.**

   The small performance gap to the fully-supervised baseline suggests that the feature encoder is able to derive relevant features without needing additional fine-tuning of the feature encoder. This performance is in line with similar zero-and few-shot research using vision encoders on other tasks. For instance, Zhou et al. demonstrated that even for difficult modalities such as remote sensing images, CLIP vision features can achieve strong performance without needing any fine-tuning [2].

3. **Currently, all the proposed prompts are annotated by humans. We need to try-and-error and go over to have the best selection among them. Is there any way to find the most adaptive prompt (or even the soft, in the features space, one as [13]) in a hands-free manner? This can make the whole system more practical and ensure further robust performance.**

   We thank the reviewer for their suggestion, and indeed we have wondered the same during experimentation. We have tried training meth-

ods such as CoOp, the method of [13], for the task of scenicness prediction. We optimized over the text prompt input space rather than the image embeddings, with our scenicness scores as a new output objective for these methods. However, this experiment was not as straightforward as in the classification setting, as CoOp did not converge for scenicness prediction. Moreover, by freely optimizing over the prompt token space, they could produce embeddings which are more accurate, but way more difficult to understand. We believe that the question of hands-free tuning is interesting, but would require efforts beyond this paper. In future work we will explore ways to make these methods work well for regression tasks, while still providing meaningful insights into the vocabulary of aesthetics. We have added those reflections to our conclusions.

Reviewer 3

The paper presents a rich variety of graphical displays. However, it lacks theoretical innovation and lacks theoretical support for the motivation between specific computational steps.

This paper evaluates the use of VLMs for landscape scenicness assessment purposes. It is indeed an applied paper bridging recent computer vision advances with more perception-based studies and should be considered as such. With this paper we are not aiming for theoretical contributions to computer vision methods, and while competition against benchmark approaches is indeed interesting and worthwhile, we do not view it as a core contribution of the work. Instead, we believe that the proposed methods will be useful for efficient, large-scale landscape quality evaluation by government agencies and NGOs.

That said, we understand the concern of the reviewer, so in this revision we run a number of extra comparisons. To further support the validity of our experiments, we have added an experiment for the prompt consistency with land cover classes, in order to provide grounded support for the late ensembling method over early ensembling. Moreover, we provide a comparison against another encoder pre-training method, SigLIP, which can be found in Appendix 1 of the paper.

In terms of experimental design, the authors should incorporate additional methods for similar tasks and provide comparative results with different backbones.

The reviewer's comment exactly addresses the core contribution of our paper: while additional tasks will be helpful in determining the transferability of the methods to similar objectives, there is a lack of reliable and openly available reference data for similar visual perception tasks. This study specifically tries to address this by investigating the possibilities provided by data-efficient learning and annotation schemes so that new datasets for similar tasks may be established. In this paper, we consider scenicness as a task for which we have ample information, but we hope that many more will appear in the near future, hopefully using our prompting method. We have added these reflections to our conclusions.

Regarding the use of backbones, we have performed comparison experiments with SigLIP-pretrained embeddings rather than CLIP embeddings, but found little effect on the performance of the methods. To avoid shifting focus from landscape scenicness perception towards model comparison, we decided to report these findings in Appendix 1 of the paper.

References

[1] Xiaohua Zhai, Basil Mustafa, Alexander Kolesnikov, and Lucas Beyer. Sigmoid Loss for Language Image Pre-Training. In *Proceedings of the 2023 IEEE/CVF International Conference on Computer Vision*, Vanves, September 2023. IEEE Computer Society. arXiv:2303.15343 [cs].

[2] Kaiyang Zhou, Jingkang Yang, Chen Change Loy, and Ziwei Liu. Learning to Prompt for Vision-Language Models. *International Journal of Computer Vision*, 130(9):2337–2348, September 2022.
